# Balancing feeling ‘prepared’ without feeling ‘devoured’: A qualitative study of self‐care from the perspective of self‐empowered persons living with Parkinson's disease in Sweden

**DOI:** 10.1111/hex.14027

**Published:** 2024-03-25

**Authors:** Jamie L. Luckhaus, Anna Clareborn, Maria Hägglund, Sara Riggare

**Affiliations:** ^1^ Department of Women's and Children's Health, Participatory eHealth and Health Data Uppsala University Uppsala Sweden; ^2^ Department of Immunology, Genetics and Pathology, Rudbeck Laboratory Uppsala University Uppsala Sweden; ^3^ Uppsala University Centre for Disability Studies Uppsala University Uppsala Sweden

**Keywords:** Parkinson's disease, qualitative, self‐care, self‐empowerment, self‐management patient author, self‐tracking

## Abstract

**Introduction:**

Parkinson's Disease (PD) is a complex neurodegenerative disease resulting in a wide range of motor and nonmotor symptoms for which the treatment regimen is often complex. People with Parkinson's (PwP) spend time daily on self‐care practices including self‐tracking signs and symptoms or seeking disease‐specific knowledge. Research suggests self‐care interventions yield promising care and health outputs for PwP, yet most research focuses on the provider perspective rather than that of those conducting the self‐care. This study explores the meaning of self‐care, disease‐specific knowledge, and self‐tracking from the perspective of PwP in Sweden.

**Methods:**

Qualitative data from three data sets were analyzed and compared using qualitative content analysis: one focus group on self‐care (*n* = 14), one free‐text survey on disease‐specific knowledge (*n* = 197) and one free‐text survey on self‐tracking (*n* = 33).

**Findings:**

The analysis resulted in three categories: *illness‐related tasks, internal resources* and *external resources*. Illness‐related tasks describe various tasks PwP carry out in self‐care, including lifestyle choices, treatments, and self‐tracking. Internal resources include personal knowledge/skills as well as mindsets which could facilitate or challenge completing these tasks. Finally, external resources include other PwP, literature, clinicians and other sources of disease‐specific knowledge. Self‐care was found to fluctuate between beneficial and burdensome depending on such resources.

**Conclusions:**

In conclusion, self‐care needs to be acknowledged and discussed more often in PD and other complex conditions. Future self‐care interventions should consider self‐tracking and disease‐specific knowledge as well as internal and external resources in their design and implementation.

**Patient or Public Contribution:**

A researcher with PD was actively involved in all phases of the research: study design, data collection and analysis, and preparing the manuscript.

## 1 INTRODUCTION

Parkinson's disease (PD) is a complex neurodegenerative condition, estimated to affect over 6 million people worldwide.^1^ People with Parkinson's disease (PwP) experience a wide range of motor and nonmotor symptoms and treatment regimens become increasingly complicated with time since onset. Treatment may include a variety of therapies (e.g., infusion therapies, transdermal patches, deep brain stimultation) but primarily include oral pharmaceuticals, where as many as five intakes daily are not uncommon. It is therefore essential that PwPs are knowledgeable and active in their treatment decisions and self‐care.^2^, ^3^

A review of PD interventions found self‐care, where PwP manage their condition (psychological, physical and practical) outside of institutionalized or professional care,^4^ to be effective in improving care and outcomes for PwP.^5^ Research has suggested both self‐monitoring/self‐tracking (e.g., logging signs, symptoms and activities on paper or an app) and disease‐specific knowledge as important aspects of self‐care.^6^, ^7^, ^8^ This is because self‐care is not only about managing one's health, but also one's *ability* to do so^9^ (e.g., through self‐tracking and applying disease‐specific knowledge). An example of how knowledge can yield positive (self‐)care outcomes comes from an interview study with PwP in Sweden, where participants described using self‐tracking to decide when to seek healthcare services.^10^ Although many terms, including self‐management, are used in reference to these practices, we will use the term *self‐care* in this paper. Self‐care requires active responsibility over day‐to‐day health management from the person living with a chronic condition.^11^ Patient empowerment is generally understood as healthcare professionals (HCPs) guiding patients in (re)gaining control over their health resulting in literature and interventions aimed at helping HCPs empower patients through facilitating self‐care. However, self‐empowered patients may also take action of their own accord to improve their self‐care independently of healthcare (some because they feel they have no choice^12^), and can thereby improve their abilities and confidence.^12^ Despite the individual nature of self‐care and the burden of responsibility placed on patients, literature mostly explores self‐care from a *provider* point of view, rather than from the person carrying out the self‐care.^13^ For example, a study among heart failure patients defines self‐care as ‘a concept that encompasses a set of health behaviors associated with improved patient outcomes (…) [including] medication taking, exercise, diet, weight measurement, symptom recognition and response and fluid management’.^14^ This definition, like other similar definitions, fails to acknowledge aspects of self‐care beyond that of interest to the HCPs.

In Sweden there are around 22,000 PwP^15^ and a majority spend 1 h or less with their neurologist annually.^16^ This means that PwPs spend the majority of their time self‐managing their health. Efforts have been taken in Sweden to increase the understanding of self‐care for PwP.^17^ It is nevertheless likely that PwP' views of self‐care, including knowledge and tracking, differ from that of HCPs.^18^ This study, therefore, aims to explore self‐care, disease‐specific knowledge, and self‐tracking from the perspective of self‐empowered PwP to better understand how people living with PD understand and relate to these concepts.

## 2 METHODS

This study is a triangulation of three data sets, drawing on qualitative data from a focus group activity and two surveys conducted over a 5‐year‐period with PwP in Sweden.

### 2.1 Data sources

Our data originates from three sources:

#### 2.1.1 Data set I

**Figure 1:**
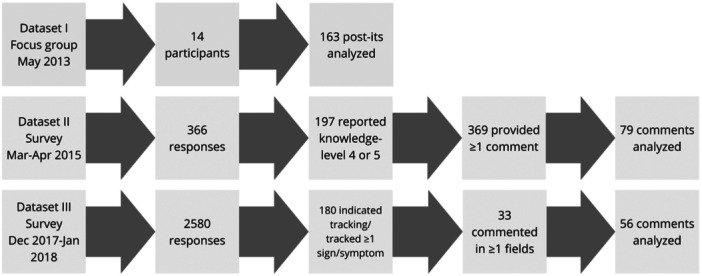
Overview of the three data sets, showing the time of origin, number of participants/responses, selection criteria, clarifications and the number of comments analyzed in this paper.

Data set was collected during part of a longer focus group on PD self‐care organized in May 2013. The focus group included 14 participants (Table 1) who were purposefully recruited from patient networks to represent self‐empowered PwP. Data consist of statements written on post‐it notes. The participants were asked ‘what does PD self‐care mean to you?’ They were instructed to individually write one statement/answer on each note and to generate as many notes as possible. In total, 163 notes were generated, all of which were included in the analysis (Figure 1).

**Table 1:** Data set I focus group participant characteristics: age and time since diagnosis.

Data set I: Self‐care of PD (*n* = 14)						
Age	Born 1920s	Born 1930s	Born 1940s	Born 1950s	Born 1960s	Born 1970s
Female *n* = 8	–	–	2	2	4	–
Male *n* = 6	–	–	2	2	2	–
Total *n* = 14	–	–	4	4	6	–
**Time since diagnosis**	<5 years	5–10 years	>10 years	–	–	‐
Female, *n*	5	2	1	–	–	–
Male, *n*	3	2	1	–	–	–
Total, *n*	8	4	2	–	–	–

Abbreviation: PD, Parkinson's disease.

#### 2.1.2 Data set II

Data set II consists of free‐text comments collected via an online survey about disease‐specific knowledge of PD from March to April 2015. The quantitative results have been reported previously^16^ but the free‐text comments were not included in the previous analysis. Of 366 responses (Table 2), 197 reported their knowledge level as being a 4 or a 5 on a 1–5 scale (seen as self‐empowered). These respondents contributed in total 369 comments, 79 of which were included in the analysis.

**Table 2:** Data set II surrey free‐text participant characteristics: Age, education level, county of residence.

**Data set II: PD‐specific knowledge (*n*** = **197)**						
**Age**	Born 1920s	Born 1930s	Born 1940s	Born 1950s	Born 1960s	Born 1970s
Female *n* = 94	0	16	44	21	10	0
Male *n* = 103	1	20	52	22	6	2
Total *n* = 197	1	36	106	43	16	2
**Time since diagnosis**	<5 years	5–10 years	>10 years	Unknown	–	–
Female *n* = 94	32	31	22	9	–	–
Male *n* = 103	30	43	30	–	–	–
Total=197	62	74	52	9	–	–
**Highest attained education level**	Primary education	Secondary education	Postsecondary (higher) education	Vocational training	Military training	Unknown/other
Female *n* = 94	9	16	61	4	0	4
Male *n* = 103	25	27	45	4	1	1
Total *n* = 197	34	43	106	8	1	6

Abbreviation: PD, Parkinson's disease.

#### 2.1.3 Data set III

Data set III consists of free‐text comments collected via an online survey about self‐tracking PD signs and symptoms from December 2017 to January 2018. The quantitative results have been reported previously^10^ but the free‐text comments were not included in the previous analysis. Of 280 respondents (Table 3), 180 indicated having tracked ≥1 sign or symptom regarding their PD, which was interpreted as an indication of self‐empowerment. Of these, 33 left at least one comment, resulting in 56 comments included in the analysis.

**Table 3:** Data set III survey free‐text participant characteristics: Age, education level, county of residence.

Data set III: self‐monitoring and self‐tracking PD (*n* = 33)					
Age	Age 36–45	Age 46–55	Age 56–65	Age 66–76	Age 76–85
Female *n* = 18	1	3	6	5	3
Male *n* = 15	0	2	2	8	3
Total *n* = 33	1	5	8	13	6
**Time since diagnosis**	<5 years	5‐10 years	>10 years	Unknown	–
Female *n* = 18	7	8	3	–	–
Male *n* = 15	2	8	5	–	–
Total *n* = 33	9	16	8	–	–
**Highest attained education level**	Primary education	Secondary education	Postsecondary (higher) education	–	–
Female *n* = 18	0	4	14	–	–
Male *n* = 15	1	5	9	–	–
Total = 33	1	9	23	–	–

Abbreviation: PD, Parkinson's disease.

See Figure 1 for an overview of data collected and analyzed. The included participants are interpreted as self‐empowered, as they self‐reported active self‐care practices, including high disease‐specific knowledge and experience of self‐tracking. Both surveys were disseminated across Sweden for anyone with a PD diagnosis, regardless of disease progression, time since diagnosis, age, gender or any other factors. It is possible that individuals participated in more than one of the data sets.

### 2.2 Data analysis

Data was analyzed through qualitative content analysis^19^ using Miro© online collaborative whiteboard.^20^ S. R. was responsible for the original data collection for all data sets and refamiliarized herself with the data in parallel with J. L. L. The analysis was led by J. L. L. with support from the other authors.

Data was transferred into Miro© as virtual post‐its. The meaning units were already short and concise, as they consisted of survey responses and post‐it notes, and therefore did not need further condensing. Due to the nature of the meaning units, codes were applied on a manifest level. Data was coded so that each code applied to multiple post‐its or meaning units.

An inductive analysis was conducted on each data set separately, resulting in preliminary categories. Second, the data sets were analyzed as a whole by comparing and combining categories, producing three overarching categories. The categorization between each iteration was documented to enhance transparency and facilitate collaboration. When the interpretation of meaning units was ambiguous, the source material for each data set was revisited to aid interpretation by looking at the exact question posed, as well as other answers given. The final fine‐tuning of categories was conducted collaboratively to maintain reflexivity by combining various perspectives^21^ including a PwP perspective. Finally, illustrative quotations were selected and translated from Swedish to English with minor adjustments to enhance comprehensibility.

## 3 FINDINGS

Analysis of the data sets highlighted that for PwP, self‐care is a broad and varied concept, which can include various resources—mindsets, knowledge, friends, clinicians, and so forth—in addition to tasks. The triangulation produced three categories: *illness‐related tasks, internal resources* and *external resources*, described below with illustrative quotations.

### 3.1 Illness‐related tasks

This category covers the various tasks participants described carrying out as part of their self‐care practices. Healthcare‐related tasks, that is, things you need a referral or prescription to access, such as medicines and certain therapies, and lifestyle choices such as diet and exercise were among the various tasks described. The exercise was the most frequently mentioned lifestyle choice, some of which involved social forms of exercise such as group training with ‘friends from PD gym’. Other forms of self‐care listed include massage, yoga, and spending time in nature. A variety of nonmedical therapies were named, including breathing exercises and brain training. Self‐care also included healing, recuperation and stress‐reduction, sometimes in the form of simple, everyday activities such as ‘petting the cat’.

Keeping track of one's lifestyle and symptoms was emphasized in all data sets, including monitoring sleep, weight, and medication intake. This included staying informed through tasks such as checking the patient portal for clinical notes, researching treatment options, and looking for symptom patterns. In data set III, one participant noted that ‘[self‐tracking] helps me understand how supplements affect my symptoms’ (male, <65) This indicates that self‐tracking and knowledge‐seeking (internal resources) are an illness‐related task in themselves and a part of self‐care.

Participants in both data sets I and III listed medicine‐tracking as self‐care, with several describing adjusting their medication according to their observations.

[tracking] helps me to detect any differences between different brands of the same substance (…) I switched [medication brand] (due to price). I gradually deteriorated. After 14 days, I understood that something was wrong when I couldn't get my index finger straight to put my contact lenses in. Thanks to my notes, I was able to go back in time and see that my deterioration coincided with the switch (…). I switched to another brand and improved. (female, 65+, data set III)

Additionally, some choose not to self‐track, as it causes stress. ‘[I] do not want to have an exact measurement of how quickly I am getting worse’ (female, 65+, data set III). Just as deciding to track yielded positive outcomes for some, others consciously refrained, which is another way of managing well‐being.

### 3.2 Internal resources

Participants mentioned various internal resources as a part of self‐care. Having a positive mindset and/or in‐depth disease‐specific knowledge and skills aids self‐care practices, whereas a lack would pose additional challenges. Obtaining these resources, as described above, was also seen as an illness‐related task.

#### 3.2.1 Knowledge‐seeking and self‐prioritization mindsets

Personal knowledge is an important internal resource for accepting and building awareness of one's condition as well as taking action. A couple of respondents were HCPs themselves, and therefore had relevant knowledge through their profession. However, others described ‘taking initiative’ by actively seeking disease‐specific knowledge and skills. ‘[I] think and ponder a lot, then compile on paper, [and] read about the disease’ (female, 65+, data set III). Several of the post‐its (data set I) included passive language where nouns were listed, for example, ‘scientific research’ although most participants used active verbs (‘search’, ‘learn’) and using information ‘to make a decision’ about whether to seek healthcare services. Self‐care is ‘daring to be “your own” doctor’, (data set I) trusting yourself, and taking an active role in your health*. ‘*Knowledge is fundamental to how I can manage my disease, how I interpret the symptoms and effect of treatments. Knowledge helps me reduce anxiety and feel safe and competent (female, 65+ data set II).

Some participants described a mindset of putting oneself first; you must have the energy and motivation to manage your condition, being ‘active even when you are tired’ (data set I). Self‐care was seen as being able to ‘listen to your body’ (data set I) and prioritize your own needs. This included learning to say ‘no’, expressing your needs, working less, and spending time on what makes you happy. This mindset was also seen in tracking participants, where they described sharing only as much of their data with HCPs as felt necessary. Reflection and mindfulness were also seen as self‐care, including alone time, journaling, practicing forgiveness, and living in the moment. ‘Try to avoid thinking about how it is going to be—live in the now’ (data set I). Self‐care was described as not only managing PD, but also about life beyond having a chronic disease. Participants expressed themes of creating normalcy. It is important to have fun and a sense of humour, and to ‘enjoy the moments in between’ (data set I). Maintaining a positive mindset, including to ‘focus on what I can do ‐ —not what I can't do’, (data set I) was seen as relevant, as well as accepting yourself and others, and creating routine and structure. A couple of participants listed shifting focus to ‘counter’ one's disease, such as through thinking about clothing and hairstyle. *‘*Get involved in something that requires attention and concerns something other than sickness. For example, painting [and] genealogy’ (data set I).

#### 3.2.2 Finding a balance of feeling ‘prepared’ without feeling ‘devoured’

Participants highlighted an array of negative and positive emotions that can result from knowing more about one's condition. On the one hand, seeking information or systematically observing symptoms can serve as a reminder that one is sick. ‘I don't self‐track because then I feel that PD is devouring me’ (female, <65, data set III), and ‘[I] don't want to have an exact measurement of how quickly I am worsening’ (data set III). Participants also referred to hypochondria and depression as outcomes of disease‐specific knowledge. *‘*[I] become depressed from too much information about my disease, which leads to my wife gathering most of the information’ (male, 65+, data set II). Self‐care involves balancing self‐managing one's condition without hyper‐focusing to a point where life revolves around the diagnosis. ‘(…) It's also hard to constantly focus on yourself and your well‐being. I need to be normal, too’ (female, 65+, data set II).

On the other hand, participants described self‐tracking as gaining a sense of control over their health. ‘It's I who should control my life, not Parkinson's!’ Participants expressed that knowledge helps you accept the disease and prepare for whatever the future may hold. ‘Knowledge makes it easier to live with the disease’ (man, 65+, data set II). According to one respondent, knowing about your condition helps you prioritize a healthy lifestyle and have the discipline to stick to your medication regime, exercise properly, and take care of yourself. *‘*It's about accepting the disease and setting aside your own time to face the disease’ (man, 65+, data set II). Respondents stressed that disease‐specific knowledge not only prepared them for managing the disease in the present, but also for whatever may come in the future. ‘The more you know about the disease you have, the more prepared you are for what may happen in the future’ (male, 65, data set II).

Another influence of self‐tracking was positive or negative reinforcement from healthcare. In some cases, extensive self‐care was practiced to pick‐up where healthcare lacked. *‘*[My neurologist is] not much help. I manage my medication myself’ (male, 65+, data set II). Many other participants described using insights from self‐tracking (internal resources) to prepare for healthcare visits and be able to inform their HCP of their situation. Respondents expressed that no one knows their condition as well as they do, as they live with it.

[The] doctor thinks he/she knows the answer but it is I who knows, for example, how long it takes for the medicine to kick‐in. The doctor says 30 minutes but I know that it takes around 50–70 minutes for me. (female, 65+, data set III)

It is not rare for medical personnel to wrongly assume they have all relevant information. By sharing the results of their self‐tracking with HCPs, patients invite them to see a fuller picture, thereby helping HCPs help them. ‘Only I know how [PD] feels for me. [I] must be able to show [my tracking results] so medical staff understand even though they do not have Parkinson's disease’ (female, 65+, data set III).

#### 3.2.3 Needing knowledge to learn

Another important aspect of internal resources is needing knowledge to learn more. It is hard to know what and/or how to track without the relevant knowledge and skills. ‘It's hard to decide what's most important to measure and how to do it effectively. [I] don't have good resources’ (female, 65+, data set III). Respondents to the self‐tracking survey said it is hard to select and prioritize variables to track, and that they do not know what methods are available. One respondent noted that they have not seen a recommended tracking method from the patient organization nor from healthcare. Another mentioned that they have high motivation to self‐track and that have even tried it, but that they do not know what to look for, nor how to draw conclusions. Another noted that they often misjudge the situation when they attempt to track in their head.

#### 3.2.4 External resources

Participants mentioned external resources, such as books, scientific literature, the internet HCPs friends, and family. These were sources for obtaining the internal resources described previously. Talking to other PwP, including family members or friends from PD meet‐ups, constituted the largest amount of data set I data in this category, which was also observed in data set II.

Regarding HCPs, nurses, physiotherapists, and the PD team were mentioned several times, while the neurologist was rarely mentioned an information source. Most comments about the neurologist concerned how pressed for time they are, regardless of whether respondents commented favourably of their PD physician or not. Several participants expressed knowledge‐seeking as a collaboration with HCPs (data sets II and III). Respondents from each survey mentioned self‐tracking to facilitate an exchange of information with HCPs. ‘[I] use [the national online healthcare portal] regularly and describe how I am doing. The doctor then gives suggestions regarding medication and I report back how I respond’ (male, 65+, data set II). There were examples of positive and negative reactions from HCPs, which reinforce or discourage continued tracking and active participation in one's care, that is, self‐care. A couple of participants also noted that the healthcare system does not provide enough information.

In addition to collaborating with HCPs, participants described reflecting on their health observations through talking to others, especially their partner, family, and friends. When asked what aspects of PD they track, some answered they even let others track for them. ‘I have a wife and friends who keep track of me’ (male, 65+, data set III) and ‘my (female) friend keeps a diary for me’ (female, 65+, data set III).

#### 3.2.5 Self‐care: An overarching concept

**Figure 2:**
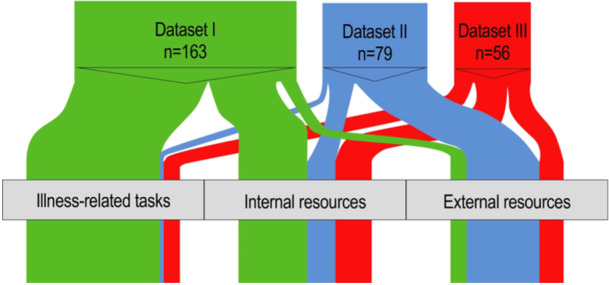
Proportional distribution of meaning units from each data set into the different categories, where *n* represents meaning units. Data set I: self‐care focus group, data set II: disease‐specific knowledge survey and data set III: self‐tracking survey.

Each category contains meaning units from all data sets, and an overview of the data distribution is presented in Figure 2. The mixture of data sets in each category suggests an overlap and interconnectedness between the three phenomena: self‐care, disease‐specific knowledge, and self‐tracking from the perspective of self‐empowered PwP. It also describes resources (including self‐tracking and disease‐specific knowledge) as *a part of* self‐care *in addition to* influencing self‐care.

To exemplify further, participants listed ‘knowledge’ and ‘seeking information’ when defining self‐care, while another participant listed ‘improved self‐care’ as motivation for seeking knowledge. Similarly, one participant explained that disease‐specific knowledge gained through self‐tracking insights also led to improved clinical visits, which can be seen as an illness‐related task. Keeping track of one's health status, medications, and lifestyle choices were defined as illness‐related tasks, although this also involved using self‐tracking methods and obtaining disease‐specific knowledge to do so. Remaining social was an illness‐related task listed in data set I, although social activities such as participating in PD groups and meeting others with PD were also mentioned as resources. Asking for input from friends and family was a way of self‐tracking with their support. Remaining social, maintaining a routine, shifting focus away from PD occasionally, as well as having thirst for knowledge, were all mindset building blocks attributed to self‐care. However, having a positive and constructive mindset is an internal resource rather than a task, putting it in line with other internal resources from data sets II and III.

## 4 DISCUSSION

In this study, we have explored the views of self‐empowered PwP in Sweden on self‐care, disease‐specific knowledge, and self‐tracking by triangulating three data sets. The main finding when data was compared and contrasted, was that there was vast overlap between the phenomena. It is clear that self‐care is on a higher, all‐encompassing level, and the three phenomena are difficult, if not impossible, to isolate. Qualitative analysis yielded three mixed‐data set categories: *illness‐related tasks, internal resources*, and *external resources*.

### 4.1 Self‐care includes resources

An interesting finding of this study was that PwP considers internal and external resources to be a *part* of self‐care. This is in contrast to common definitions of self‐care and self‐management, where self‐care entails the tasks HCPs assign patients rather than HCPs supporting patients in work the patients themselves consider important.^22^ Additionally, our analysis found overlap in responses between data sets, indicating that self‐tracking, and other forms of obtaining disease‐specific knowledge, were seen as interconnected with self‐care. This aligns with Nunes & Fitzpatrick's definition where ‘[self‐care] includes activities such as observing changes in the body’ and ‘acting on symptoms. From the perspective of someone with a chronic illness, self‐care is far more than health‐care‐related tasks, but also the resources which impact those tasks. Tracking and reacting to health and treatment, managing psychological and practical implications are parts of self‐care. As our participants described, knowledge is empowering, but sometimes ignorance is bliss. Self‐care is a balance of all illness‐associated activities and maintaining well‐being in the presence of illness.

Lorig and Holman^23^ proposed ‘five core self‐management skills: problem solving, decision making, resource utilization, forming of a patient/healthcare professional partnership, and taking action’, each of which was reflected in our data. Participants described tracking when they noticed a health problem or wanted to increase their awareness. They also described using knowledge for decision‐making and saw this as part of self‐care. Resource utilization is a key part of self‐care according to our data, and can be seen as both a skill and self‐care practice. Varying relationship dynamics with an HCP were also described in our data, where some experienced positive, and others negative reinforcement from their HCPs in response to self‐tracking.

#### 4.1.1 Socioeconomic considerations

With resources being a crucial factor for self‐care, consideration of socioeconomic differences is paramount. More women in our sample have a secondary education than men. There were nearly as many females as males, although PD affects more men than women,^24^ suggesting that women may be more active in research and/or self‐care practices. Multiple male participants expressed that their wives played a large part in managing their PD, and others mentioned family/friends. Socioeconomic status likely impacts the informal caregiver support one has.

#### 4.1.2 Technological advances and patient movements

It is important to consider how digital and societal advances from data collection I (2013) to III (2017) may have impacted the results.

Self‐care was seen as influenced by health literacy and informants listed their information sources, where the internet was mentioned about as frequently as nondigital methods. Perhaps if surveyed today, the internet and technologies (e.g., mHealth) would dominate responses. The most recent data set focuses on self‐tracking, which became mainstream as wearables and other technological advances became widely available, possibly influencing responses. However, the general importance of pattern‐seeking and methods of using informal caregivers likely transcend tracking methods. Additionally, this study sample, like the population of PwP, is mostly above age 65 and perhaps less comfortable using new technologies.

Technological advances are not apparent in the data sets, though there is a noticeable shift in agency. Data set I describes activities and mindsets that are unclear as to the level of HCP involvement. Data set II included using disease‐specific knowledge to collaborate with HCPs. Self‐tracking (data set III) included using self‐tracked information to act independently of HCPs, such as to adjust medications and to inform their HCPs. This may be due to the nature of the questions, or possibly an influence of patient organizations and healthcare gradually redefining the patient role, abandoning the view of the patient as a passive recipient of healthcare, and adopting shared decision‐making.^25^

### 4.2 The burden of self‐care fluctuates

Our findings align with aspects of Tran et al.'s ‘burden of treatment’^26^ for chronic conditions, in which one burden is ‘healthcare tasks’, including ‘lifestyle changes’ and ‘management of medications’. However, our findings suggest that illness‐related tasks, including lifestyle and medication choices, are not a burden per se, as this depends on resources and perspective of the individual, and likely fluctuates over time. Some of the tasks, such as observing one's health, were described as empowering and as improving (self‐)care, but also as causes for worry. The associated burden has more to do with one's mindset and knowledge (internal resources), as well as one's support system (external resources). Self‐care can be done in isolation, but is more commonly a collaboration between the patient, informal caregivers and HCPs.^27^ This further highlights the importance of the external resources we identified, as the quality of these resources greatly impacts quality of self‐care, and whether it feels burdensome.

Another concept of interest is Van Bulck et al.'s ‘illness identity’, described as ‘the degree to which a chronic health condition (…) is integrated into someone's identity’, where one's identity was found to impact level and effectiveness of self‐care.^28^ Composed of four ‘identities’: engulfment, rejection, acceptance, and enrichment, our data reflected aspects of engulfment, as well as expressions of acceptance and enrichment.^28^ Perhaps the latter two are unsurprising, as our participants were considered self‐empowered patients who had self‐tracked and participated in research. However, the fact that the same participants at times expressed negative as well as positive experiences of self‐care, suggests that one can belong to multiple illness identities throughout time shifting from one to another, rather than adopting a set ‘identity’. Once again, this shift may be related to resources rather than an individual's set ‘identity’.

Additionally, our findings suggest that many of these tasks are related to healthcare to varying degrees, but may also be healthcare independent. For example, exercising, socializing with other PwP, working less and practicing mindfulness may all be recommended by a HCP, but could also be completely at the initiative of the PwP and never discussed with an HCP. Some of our participants described practicing self‐care tasks without their HCP, and in at least a couple of instances due to a lack of interest and/or knowledge provided by their HCP. We, therefore, found ‘illness‐related tasks’ to be more suitable than ‘healthcare tasks’.^26^

### 4.3 Self‐empowerment in self‐care

This study's participants were all active in their own health management independently of healthcare—that is, self‐empowered. A large portion of the activities listed as self‐care were activities not mandated by HCPs. Additionally, participants described feeling more in control or relaxed thanks to knowledge about their condition. Aujoulat et. al. argue that in regard to patient empowerment ‘the process of relinquishing control is as central to empowerment as is the process of gaining control’.^29^

Health literacy reflects a similar phenomenon to self‐empowerment, in the sense that knowledge in and of itself does not yield literacy, one must *apply* said knowledge.^30^ Anexample of a valuable external resource which requires health literacy is the patient electronic health records (EHRs). Accessing one's EHR was listed in our study as an illness‐related task contributing to self‐tracking and remaining in the know about one's PD. The sharing of clinical notes with patients came only recently in many nations,^31^ though research suggests patient‐accessible EHRs are a valuable resource that increases patient autonomy and facilitates co‐production of care.^32^ This resource highlights the importance of digital health literacy, a subset of health literacy important in our increasingly digitized society.^33^

Even the ‘expert’ patients in our study expressed challenges with not knowing how or what to track, obtaining relevant information, and the negative effects of being active in self‐care. It is important to consider that these challenges are likely greater among less active patients, as disparities in health literacy are vast.^34^ Additionally, our results demonstrate that empowerment, literacy and enrichment are not a fixed status that one achieves and never leaves. Our participants, like anyone with chronic illness, experience ups and downs and need support from healthcare and patient organizations.

### 4.4 Strengths and limitations

Surveys and post‐its only allow short, concise responses and do not capture the same depth as interviews. However, this allowed gathering a larger sample size, making it possible to include a larger variety of perspectives. Also, because responses were anonymous and written rather than communicated face to face, participants may have felt more comfortable sharing opinions that they might not have voiced otherwise. Data set I is from around 10 years ago. However, the topic and responses are still relevant, and this data set was triangulated with more recent data sets. Having a longitudinal aspect coupled with a large sample size gives strength to the data.

In regard to transferability, online surveys and focus groups tend to be attended by patients already active in their own healthcare. As we were interested in the perspective of self‐empowered patients, this was not a problem. The transferability to other countries is unknown. However, factors such as the time and frequency spent with a neurologist have been reported to aid others in judging transferability in regard to these care aspects. Triangulation of data sources is also a strength for transferability.^35^

One of the researchers (S. R.) carries a dual perspective as she has PD herself, has conducted self‐tracking^36^ and been part of developing frameworks for patients' self‐empowerment.^8^, ^12^ We are, therefore, aware that there could be bias in the analysis, however, we have mitigated this risk by having another researcher conduct the preliminary analysis. Additionally, we felt the value added from having the perspective of someone with PD in each step of the study outweighed the challenges. All authors have prior experience and training in qualitative data collection and analysis, as well as experience researching self‐care for chronic conditions.

## 5 CONCLUSIONS

Our qualitative study exploring self‐care, disease‐specific knowledge and self‐tracking from the perspective of self‐empowered PwP in Sweden, found that these phenomena are intertwined and difficult to isolate. Self‐care involves utilizing resources to carry out illness‐related tasks. Furthermore, participants expressed a fluctuation between burden and benefit when practicing self‐care. It is important to consider how resources impact self‐care, and that challenges identified in our study may be graver among disadvantaged groups. Assisting PwP in identifying valuable parameters and viable methods for self‐tracking, taking a person‐centred approach, is an example of how healthcare teams and patient organizations can help PwP find an individually‐suited self‐care routine.

In conclusion, self‐care needs to be acknowledged and discussed more often in PD and other complex conditions. Future self‐care interventions should consider self‐tracking and disease‐specific knowledge as well as internal and external resources in their design and implementation. Additionally, it is important to continue to explore these terms from the perspective of persons living with chronic conditions.

## AUTHOR CONTRIBUTIONS

**Jamie L. Luckhaus**: Methodology; formal analysis; writing—original draft; visualization; writing—review and editing. **Anna Clareborn**: Methodology; writing—original draft; writing—review and editing; validation. **Maria Hägglund**: Conceptualization; methodology; writing—review and editing; validation. **Sara Riggare**: Conceptualization; methodology; data curation; supervision; writing—original draft; investigation; visualization; writing—review and editing; validation.

## CONFLICT OF INTEREST STATEMENT

The authors declare no conflicts of interest.

## ETHICS STATEMENT

Participants in the focus group (data set I) were informed and provided written consent before the focus group. Ethical approval was given by the regional ethical review board in Stockholm (decision 2012/1911‐31/5). The surveys of data sets II and III were exempted from ethical approval by the regional ethical review board in Stockholm (according to decision 2015/1572‐31/4). 

## Data Availability

The data that support the findings of this study are available on request from the corresponding author. The data are not publicly available due to privacy or ethical restrictions.
